# A retrospective analysis of myocardial preservation techniques during coronary artery bypass graft surgery: are we protecting the heart?

**DOI:** 10.1186/s13019-014-0184-7

**Published:** 2014-12-31

**Authors:** Luciano Candilio, Abdul Malik, Con Ariti, Sherbano A Khan, Matthew Barnard, Carmelo Di Salvo, David R Lawrence, Martin P Hayward, John A Yap, Amir M Sheikh, Christopher G A McGregor, Shyam K Kolvekar, Derek J Hausenloy, Derek M Yellon, Neil Roberts

**Affiliations:** The Hatter Cardiovascular Institute, University College London, London, UK; The Nuffield Trust, London, UK; The Heart Hospital, University College London Hospital, London, UK

**Keywords:** Coronary artery bypass graft surgery, Peri-operative myocardial injury, Antegrade cardioplegia, Retrograde cardioplegia, Cross-clamp fibrillation

## Abstract

**Background:**

Retrograde perfusion into coronary sinus during coronary artery bypass graft (CABG) surgery reduces the need for cardioplegic interruptions and ensures the distribution of cardioplegia to stenosed vessel territories, therefore enhancing the delivery of cardioplegia to the subendocardium. Peri-operative myocardial injury (PMI), as measured by the rise of serum level of cardiac biomarkers, has been associated with short and long-term clinical outcomes. We conducted a retrospective analysis to investigate whether the combination of antegrade and retrograde techniques of cardioplegia delivery is associated with a reduced PMI than that observed with the traditional methods of myocardial preservation.

**Methods:**

Fifty-four consecutive patients underwent CABG surgery using either antegrade cold blood cardioplegia (group 1, n = 28) or cross-clamp fibrillation (group 2, n = 16) or antegrade retrograde warm blood cardioplegia (group 3, n = 10). The study primary end-point was PMI, evaluated with total area under the curve (AUC) of high-sensitivity Troponin-T (hsTnT), measured pre-operatively and at 6, 12, 24, 48 and 72 hours post-surgery. Secondary endpoints were acute kidney injury (AKI) and inotrope scores, length of intensive care unit (ICU) and hospital stay, new onset atrial fibrillation (AF) and clinical outcomes at 6 weeks (death, non-fatal myocardial infarction, coronary artery revascularization, stroke).

**Results:**

There was evidence that mean total AUC of hsTnT was different among the three groups (P = 0.050). In particular mean total AUC of hsTnT was significantly lower in group 3 compared to both group 1 (-16.55; 95% CI: -30.08, -3.01; P = 0.018) with slightly weaker evidence of a lower mean hsTnT in group 3 when compared to group 2 (-15.13; 95% CI -29.87, -0.39; P = 0.044). There was no evidence of a difference when comparing group 2 to group 1 (-1.42,; 95% CI: -12.95, 10.12, P = 0.806).

**Conclusions:**

Our retrospective analysis suggests that, compared to traditional methods of myocardial preservation, antegrade retrograde cardioplegia may reduce PMI in patients undergoing first time CABG surgery.

**Electronic supplementary material:**

The online version of this article (doi:10.1186/s13019-014-0184-7) contains supplementary material, which is available to authorized users.

## Background

The prompt delivery of cardioplegic solution to all regions of the heart during cardiac surgery is essential for successful myocardial protection [1]. PMI, detected through serial measurements of specific serum biomarkers including troponin I (TnI) or T (TnT) or creatine kinase (CK)-MB, is associated with worse clinical outcomes [2]-[7].

Antegrade delivery of cardioplegic solution to myocardial cells is adequate when supplied by unobstructed coronary arteries but may not be equally effective in the presence of occluded or stenosed arteries as in the case of coronary artery disease (CAD), which may lead to maldistribution of cardioplegic solution [8]. This might induce PMI and poor recovery of left ventricular function (LV) following revascularization [9]. Limitations associated with antegrade delivery may be circumvented by the administration of cardioplegic solution in a retrograde fashion through the coronary sinus which relies on the principle that coronary venous systems free of disease and valves can serve as a conduit for the delivery of cardioplegic solution in a homogenous manner [10]. With this technique, the cardioplegic solution is distributed to the cardiac microstructure through a transmural network of veins that is independent to flow-limiting lesions [11]. Nevertheless, retrograde cardioplegia presents important potential limitations, which could in part explain the reason why its use remains still relatively limited:

1. the anterior cardiac veins supplying the right ventricle (RV) are not directly connected to the coronary sinus and this may lead to a suboptimal distribution of the cardioplegic solution to the RV [12],[13];
2. accurate cannulation of the coronary sinus is crucial as failure in this might lead to the distribution of the cardioplegic solution to the right atrium and not to the venous system;
3. the perfusion pressure requires very close monitoring, as too low a pressure suggests misplacement of the cannula, and too high a pressure can cause rupture of the coronary sinus [14],[15]. These potential issues can generally be avoided by care and precision by the surgeon;
4. the delay in arresting the heart due to slow retrograde perfusion if retrograde cardioplegia is used alone (lower flow rates and pressures used to prevent coronary sinus damage and myocardial oedema) [16],[17].

Therefore, the combination of both methods of antegrade and retrograde cardioplegia is thought to overcome limitations inherent to both techniques [18]. However, although both antegrade perfusion and retrograde coronary sinus perfusion have been studied experimentally and used clinically in patients undergoing CABG surgery, there is little information comparing PMI magnitude between different methods of cardioprotection during revascularization. In this regards, we conducted a retrospective subgroup analysis to determine whether the combination of antegrade and retrograde cardioplegia is associated with improved myocardial preservation in patients undergoing CABG surgery.

## Methods

### Study design

We conducted a retrospective analysis of patients undergoing first time CABG surgery recruited between December 2010 and July 2012 as a subgroup of control patients in a large parallel single-blinded randomised controlled clinical trial carried out at the Heart Hospital, University College London Hospital (London, UK), and investigating the effects of remote ischaemic preconditioning (RIPC) [4],[5] in patients undergoing cardiac surgery. The surgery was carried out in accordance with the University College London Hospital NHS Trust guidelines and received local Ethics Committee approval. We obtained written informed consent from all patients recruited into the study.

### Inclusion and exclusion criteria

Patients included in the current analysis were recruited within a single centre study investigating the effects of RIPC [19],[20] in patients undergoing CABG surgery at the Heart Hospital (UCLH London, UK): only patients receiving the control protocol were included.

Patient exclusion criteria comprised:

- cardiogenic shock or cardiac arrest preceding surgery;
- positive baseline serum hsTnT;
- pregnancy;
- significant peripheral arterial disease (PAD) affecting upper and/or lower limbs;
- significant hepatic dysfunction (International Normalised Ratio > 2.0);
- significant pulmonary disease (Forced Expiratory Volume-1 < 40% predicted);
- renal failure with an estimated glomerular filtration rate <30 mL/min/1.73 m^2^;
- concomitant therapy with glibenclamide or nicorandil, as these medications have been demonstrated to potentially interfere with RIPC.

### Surgical procedure

Temazepam 10-20 mg was given one hour prior to surgery. Anaesthesia induction was obtained under cardiac monitoring with combinations of midazolam, etomidate, propofol, fentanyl and anti-nicotinic agents (rocuronium, vecuronium or pancuronium). Endotracheal intubation was achieved and mechanical ventilation commenced with oxygen with or without air. For anaesthesia maintenance volatile anaesthetic agents and propofol infusion with or without fentanyl were used. Continuous monitoring of arterial blood pressure, central venous pressure, nasopharyngeal temperature was carried out.

Standard non-pulsatile cardiopulmonary bypass (CPB) was initiated with the use of membrane oxygenator and cardiotomy suction, followed by the construction of all coronary artery bypass grafts, using either blood cardioplegia or intermittent cross-clamp fibrillation.

*Group 1* had 1 litre of cold blood cardioplegia given once the cross clamp was placed and maintenance cold blood cardioplegia was given down the grafts in occluded arteries and also into the aortic root every 20-30 minutes.

*Group 2* had coronary artery surgery with the cross clamp fibrillation technique and therefore no cardioplegic solution was given.

*Group 3* had warm blood cardioplegia delivered with antegrade and retrograde techniques and was performed by one cardiothoracic surgeon, with an initial 800 ml dose of antegrade cardioplegia followed by 400 ml of retrograde cardioplegia. After this, maintenance was achieved with 100 ml of retrograde cardioplegia after each anastamosis. A hot shot of warm blood without potassium was given after the internal mammary artery (IMA) anastamosis and prior to removal of the cross clamp. All anastomoses were constructed with the single clamp technique. Systemic temperature in group 3 was 35°C.

Following anastomosis of the grafts, CPB was discontinued and protamine was used to achieve heparin reversal.

### Objectives

To determine whether the addition of retrograde cardioplegia to standard antegrade cardioplegia can reduce PMI and subsequently improve short-term clinical outcomes in patients undergoing first time CABG surgery compared to patients receiving either standard antegrade cardioplegia alone or cross-clamp fibrillation.

### Study endpoints

The primary study end-point was PMI, measured by total 72 hour AUC of hsTnT. Blood samples for hsTnT were taken pre-operatively and at 6, 12, 24, 48 and 72 hours post-surgery; hsTnT was measured quantitatively by a one-step enzyme immunoassay based on electrochemiluminescence technology (Roche, Switzerland). The lower detection limit of this assay is 1 ng/L with a threshold of ≥14 ng/L indicating significant myocardial necrosis.

Secondary end-points included:

1. AKI score, classified as grade 1, 2 or 3 based on a combination of laboratory (serum creatinine levels) and clinical (urine output) parameters [21]. Serum creatinine was measured pre-operatively and 24, 48 and 72 hours post-surgery.
2. Inotrope requirement during the first 3 post-operative days, measured using the formula modified from a study by Ko et al. [22]: Inotrope score = Dosages (μg/kg/min) of:
3. Length of ICU and hospital stay;
4. New onset of AF in the three post-operative days;
5. Clinical outcomes at 6 weeks (death, non-fatal myocardial infarction, coronary artery revascularization, stroke).

### Statistical analysis

Continouos data are presented as mean (standard deviation (SD)) or median (IQR). Categorical data are shown as number (percent). The exposure variable was a categorical variable with three groups corresponding to each of the cardioprotection categories. Comparison between exposure groups was made by including the exposure variable as a categorical variable in a linear regression model for approximately normally distributed endpoint variables. For very skewed endpoint variables the median T-test was used. Where continuous endpoint variables were measured over time a repeated measures linear regression model was fitted to measure the association between exposure variable and endpoint. The assumptions of the linear regression models were performed by analysis of residuals. Categorical data were analysed using Fisher’s exact test. No adjustment for multiplicity has been made. Data were analysed using Stata version 12.1.

## Results

Included patients were recruited into an original RIPC trial enrolling a total of 180 subjects of which 90 patients were randomised to control: 36 patients were subsequently excluded (1 patient died intra-operatively and the remaining 35 underwent CABG and valve surgery or valve surgery alone). Therefore we ultimately analysed data for 54 patients undergoing CABG surgery alone: 28 received antegrade cold blood cardioplegia (group 1), 16 patients received cross-clamp fibrillation (group 2) and 10 antegrade retrograde warm blood cardioplegia (group 3).

Group 3 had a lower rate of positive family history of CAD and previous percutaneous coronary intervention (p = 0.047) whereas group 3 had a higher incidence of cerebro-vascular accidents prior to CABG surgery (p = 0.025, Table 1): no other significant difference was found among the three groups with respect to baseline patient baseline characteristics (Table 1). Expectedly, cross-clamp time was significantly lower in group 2 (p < 0.001), however all the remaining parameters of surgery were similar amongst the 3 groups (Table 1).

**Table 1:** **Patient baseline characteristics**

Patients	Group 1	Group 2	Group 3
	Antegrade cardioplegia	Cross-clamp fibrillation	Antegrade retrograde cardioplegia
	(n = 28)	(n = 16)	(n = 10)
**Age**	67 ± 8	62 ± 10	69 ± 9
**Gender**			
Male	23 (82%)	12 (75%)	8 (80%)
Female	5 (18%)	4 (25%)	2 (20%)
**Ethnicity**			
Caucasian	21 (75%)	13 (81%)	9 (90%)
Afro-Caribbean	1 (4%)	1 (6%)	1 (10%)
Asian	5 (18%)	2 (13%)	0 (0%)
Chinese	1 (4%)	0 (0%)	0 (0%)
**BMI**	27.6 ± 4.9	30.4 ± 5.0	27.8 ± 4.5
**SBP** ***(mmHg)***	131.3 ± 20.8	128.3 ± 16.9	128.9 ± 21.2
**DBP** ***(mmHg)***	70.3 ± 7.4	73.4 ± 10.6	69.1 ± 11.6
**HR** ***(bpm)***	66.8 ± 11.1	68.6 ± 10.5	65.7 ± 13.9
**Smoking history**			
Smoker	4 (14%)	4 (25%)	1 (10%)
Non-smoker	6 (21%)	4 (25%)	4 (40%)
Ex-smoker	18 (64%)	8 (50%)	5 (50%)
**Family history of IHD**	22 (79%)	13 (81%)	4 (40%)
**NYHA class**			
0	2 (7%)	3 (21%)	2 (22%)
I	8 (29%)	5 (34%)	4 (44%)
II	17 (61%)	5 (34%)	2 (22%)
III	1 (4%)	1 (7%)	1 (11%)
IV	0 (0%)	0 (0%)	0 (0%)
**CCS Class**			
0	5 (18%)	2 (14%)	3 (33%)
I	4 (14%)	5 (36%)	0 (0%)
II	16 (57%)	6 (43%)	4 (44%)
III	2 (7%)	1 (7%)	1 (11%)
IV	1 (4%)	0 (0%)	1 (11%)
**LVEF**			
>50%	19 (68%)	12 (75%)	7 (70%)
30%-50%	8 (29%)	3 (19%)	3 (30%)
<30%	1 (4%)	1 (6%)	0 (0%)
**Co-morbidities**			
Diabetes mellitus	11 (39%)	6 (38%)	0 (0%)
Hypertension	25 (89%)	13 (81%)	7 (70%)
Hypercholesterolemia	25 (89%)	15 (94%)	8 (80%)
Atrial fibrillation	3 (11%)	0 (0.0%)	2 (20%)
Previous MI	13 (47%)	3 (19%)	6 (60%)
Previous PCI	8 (29%)	1 (6%)	0 (0%)
Previous CVA/TIA	3 (11%)	0 (0%)	3 (30%)
Previous cardiac surgery	0 (0%)	0 (0%)	0 (0%)
Peripheral arterial disease	3 (11%)	1 (6%)	2 (20%)
**Drug history**			
Aspirin	3 (11%)	2 (14%)	0 (0%)
Clopidogrel/Prasugrel	2 (7%)	0 (0%)	0 (0%)
Warfarin	1 (4%)	0 (0%)	0 (0%)
Beta-blocker	22 (79%)	11 (79%)	8 (80%)
Calcium channel blocker	12 (43%)	3 (21%)	4 (40%
Statin	26 (93%)	13 (93%)	10 (100%)
ACE-I/ARB	20 (71%)	8 (57%)	6 (60%)
Long acting nitrates	7 (25%)	2 (14%)	3 (30%)
Antidiabetics			
Insulin	4 (14%)	2 (14%)	0 (0%)
Biguanide	2 (7%)	1 (7%)	0 (0%)
Sulphonylurea	5 (18%)	2 (14%)	0 (0%)
Diuretics	7 (25%)	4 (29%)	2 (20% )
**Indication for surgery**			
Angina	23 (82%)	13 (81%)	7 (70%)
Myocardial infarction	5 (18%)	3 (19%)	3 (30%)
**EuroSCORE**	3.2 ± 1.9	2.7 ± 1.7	3.8 ± 1.8
Additive perioperative risk			
Low (EuroSCORE 0-2)	13 (46%)	8 (50%)	1 (10%)
Medium (EuroSCORE 3-5)	12 (43%)	7 (44%)	7 (70%)
High (EuroSCORE >5)	3 (11%)	1 (6%)	2 (20%)
Bypass-time (min)	93.9 ± 34.6	77.2 ± 22.0	80.3 ± 12.8
**Cross-clamp time (min)**	62.2 ± 24.4	33.0 ± 7.5	63.7 ± 13.4
**Number of grafts**			
One	1 (4%)	0 (0.0%)	0 (0.0%)
Two	9 (32%)	3 (19%)	1 (10.0%)
Three	10 (36%)	10 (63%)	8 (80.0%)
Four	8 (29%)	3 (19%)	1 (10.0%)
**Anesthetic agents**			
***Induction***			
Anti-nicotinic agents			
Rocuronium	24 (86%)	12 (92%)	6 (60%)
Pancuronium	3 (11%)	1 (7%)	2 (22%)
Vecuronium	1 (4%)	0 (0.0%)	1 (11%)
Midazolam	12 (43%)	7 (54%)	6 (67%)
Etomidate	1 (4%)	2 (14%)	2 (22%)
Fentanyl	28 (100%)	14 (100%)	9 (100%)
Propofol	27 (96%)	12 (86%)	7 (78%)
***Maintenance***			
Propofol	28 (100%)	14 (100%)	9 (100%)
Volatile anesthetics			
Isoflurane	25 (89%)	13 (93%)	9 (100.%)
Sevoflurane	3 (11%)	1 (7%)	0 (0.0%)
Intra-operative GTN	24 (89%)	14 (86%)	8 (89%)

BMI = body mass index; SBP = systolic blood pressure; DBP = diastolic blood pressure; HR = heart rate; IHD = ischaemic heart disease; NYHA = New York Health Association; CCS = Canadian Cardiovascular Society; LVEF = left ventricular ejection fraction; MI = Myocardial infarction; PCI = Percutaneous coronary intervention; CVA = Cerebrovascular accident; TIA = Transient ischaemic attack; ACE-I = Angiotensin-converting enzyme inhibitor; ARB = Angiotensin receptor blocker; GTN = glyceryl trinitrate.

The following parameters have missing values: BMI (Group-2 2), SBP (Group-2 2), DBP (Group-2 2) HR (Group-2 2), Cardio-pulmonary bypass time time (Group-2 2), Cross-clamp time (Group-2 3), NYHA (Group-2 2, Group-3 1), CSS (Group-2 2, Group-3 1), Aspirin (Group-2 2), Clopidogrel (Group- 2), Warfarin (Group-2 2), Beta-blockers (Group-2 2), Calcium-channel blockers (Group-2 2), Long-acting (Group-2 2), Statins (Group-2 2), ACE-I/ARB (Group-2 2), Insulin (Group-2 2), Sulphonylurea (Group-2 2), Biguanide (Group-2 2), Diuretics (Group-2 2), Anti-nicotinic agents (Group-2 3, Group-3 1), Midazolam (Group-2 3, Group-3 1), Etomidate (Group-2 2, Group-3 1), Fentanyl-induction (Group-2 2, Group-3 1), Proprofol-induction (Group-2 2, Group-3 1), Proprofol-maintanance (Group-2 2, Group-3 1), Volatile anaesthetics (Group-2 2, Group-3 1), Intra-operative GTN (Group-3 1).

Significance difference: Family history of IHD: p = 0.041; Previous PCI: p = 0.047, Previous CVA/TIA: p = 0.025, Cross-clamp time: p = <0.001.

### Primary endpoint

**Figure 1:**
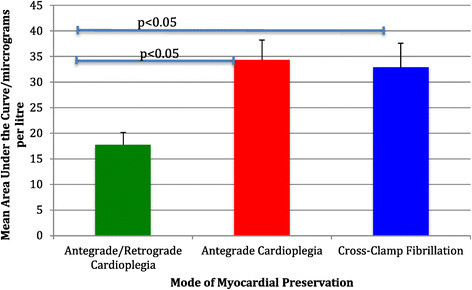
**Mean hsTnT pre-operatively and at 6, 12, 24, 48 and 72 hours post-surgery in the three study groups.**

Overall there was evidence that the mean total 72 hr AUC hsTnT was different among the three groups (P = 0.050). Examining the subgroup differences showed evidence of lower mean hsTnT in group 3 compared to group 1 (-16.55; 95% CI: -30.08, -3.01; P = 0.018) with slightly weaker evidence of a lower mean hsTnT in group 3 when compared to group 2 (-15.13,; 95% CI -29.87, -0.39; P = 0.044). There was no evidence of a difference when comparing group 2 to group 1 (-1.42,; 95% CI: -12.95, 10.12, P = 0.806) (Figure 1, Table 2).

**Table 2:** **Total hsTnT release and mean hsTnT at the 6 time-points**

Patients	Group 1	Group 2	Group 3		Comparison	Difference	Sub-group
	Antegrade cardioplegia	Cross-clamp fibrillation	Antegrade retrograde cardioplegia		Group	(95% CI)	P value
	(n = 28)	(n = 16)	(n = 10)				
	(Mean [SD])	(Mean [SD])	(Mean [SD])				
Total 72 hours AUC	34.304 (20.352)	32.885 (18.771)	17.756 (7.542)	P = 0.050	1 vs 2	−1.419 (-12.954, 10.115)	0.806
					1 vs 3	−16.548 (-30.082, -3.014)	0.018
					2 vs 3	−15.129 (-29.867, -0.390)	0.044
Pre-operatively	0.021 (0.020)	0.022 (0.022)	0.014 (0.012)	P = 0.997	1 vs 2	0.000 (-0.188, 0.188)	
					1 vs 3	−0.008 (-0.229, 0.213)	
					2 vs 3	−0.008 (-0.250, 0.234)	
6 hours post-operatively	0.961 (0.619)	0.696 (0.235)	0.399 (0.117)	P < 0.001	1 vs 2	−0.265 (-0.453, -0.077)	
					1 vs 3	−0.562 (-0.783, -0.341)	
					2 vs 3	−0.297 (-0.539, -0.055)	
12 hours post-operatively	0.752 (0.494)	0.631 (0.195)	0.319 (0.112)	P < 0.001	1 vs 2	−0.121 (-0.309, 0.067)	
					1 vs 3	−0.433 (-0.654, -0.212)312	
					2 vs 3	(-0.557, -0.070)	
24 hours post-operatively	0.508 (0.309)	0.476 (0.305)	0.266 (0.134)	P = 0.100	1 vs 2	−0.032 (-0.220, 0.156)	
					1 vs 3	−0.241 (-0.463, -0.020)	
					2 vs 3	−0.209 (-0.451, 0.033)	
48 hous post-operatively	0.359 (0.224)	0.410 (0.339)	0.227 (0.114)	P = 0.335	1 vs 2	0.044 (-0.144, 0.233)	
					1 vs 3	−0.139 (-0.360, 0.083)	
					2 vs 3	−0.183 (-0.425, 0.059)	
72 hours post-operatively	0.347 (0.231)	0.381 (0.271)	0.186 (0.119)	P = 0.257	1 vs 2	0.027 (-0.161, 0.216)	
					1 vs 3	−0.168 (-0.390, 0.054)	
					2 vs 3	−0.195 (-0.437, 0.047)	

SD = standard deviation; hsTnT = high sensitivity Troponin T; AUC = Area under the curve.

The following secondary endpoints had missing values: hsTnT 48 hours post-operatively (Group-1 1), hsTnT 72 hours post-operatively (Group-1 1), AUC (Group-1 1).

### Secondary endpoints

**Figure 2:**
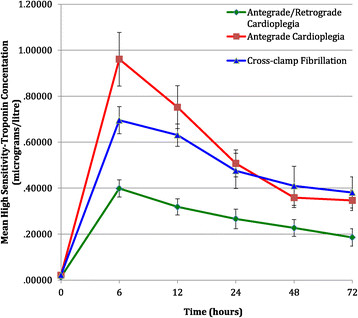
**AUC of hsTnT over the 72 hours post-surgery in the three study groups.**

Baseline pre-operative hsTnT levels were <0.02 μg/L and not significantly different between the 3 groups (Figure 2, Table 2). Overall there was evidence that the mean hsTnT differed at 6 hours (P < 0.001) and 12 hours (P < 0.001). At 6 hours there was evidence that mean hsTnT was lower in group 3 than groups 1 (-0.56; 95% CI: -0.78, -0.34). Similarly at 12 hours there was some evidence that mean hsTnT was lower in group 3 than group 1 (-0.43, 95% CI: -0.65, -0.21) (Figure 2, Table 2).

There was no evidence of a significant difference among the three groups with regards to each of the secondary endpoints (Table 3).

**Table 3:** **Summary of study secondary endpoints**

Patients	Group 1	Group 2	Group 3	P value
	Antegrade cardioplegia	Cross-clamp fibrillation	Antegrade retrograde cardioplegia	
	(n = 28)	(n = 16)	(n = 10)	
	(Mean [SD])	(Mean [SD])	(Mean [SD])	
***AKI (N)***				
0	22 (79%)	11 (69%)	10 (100.0%)	
1	5 (18%)	2 (13%)	(0.0%)	
2	0 (0.0%)	2 (13%)	(0.0%)	
3	1 (4%)	1 (6%)	(0.0%)	
Total number of AKI cases	6 (21%)	5 (31%)	0 (0.0%)	0.281*
***Inotrope score (mg/kg/hr)***				
Post bypass	7.24 (13.79)	4.03 (11.92)	3.76 (4.35)	0.816**
24 hours post-operatively	11.90 (21.32)	10.11 (22.88)	3.67 (4.36)	0.635**
48 hours post-operatively	8.76 (15.39)	14.93 (33.57)	1.94 (2.98)	0.101**
72 hours post-operatively	1.85 (5.30)	13.54 (33.13)	2.13 (2.95)	0.015**
Total	29.88 (49.10)	42.54 (94.66)	11.66 (13.79)	0.545***
***New onset AF (N)***	6 (21%)	5 (31%)	4 (40.0%)	0.475*
***Length of ICU stay (days)***	2.0 (2.0-4.0)****	3 (1.0-7.5)****	2.0 (1.0-3.0)****	0.802*****
***Length of hospital stay (days)***	8.5 (7.0-11.50)****	8.0 (6.0-10.50)****	7.5 (6.0-9.00)****	0.523*****
***Clinical outcomes at 6 weeks (N)***				
Death	3 (14%)	0 (0.0%)	0 (0.0%)	0.283*
Myocardial infarction	1 (4%)	0 (0.0%)	0 (0.0%)	1.000*
Stroke	0 (0.0%)	0 (0.0%)	0 (0.0%)	1.000
Revascularization	0 (0.0%)	0 (0.0%)	0 (0.0%)	1.000*

SD = standard deviation AKI = Acute Kidney Injury; AF = atrial fibrillation; ICU = Intensive Care Unit.

*P value for Fisher’s exact test.

**P-value from repeated measures linear regression model.

***P-value form linear regression model.

****Results shown as median (inter-quartile range).

*****P-value for Median T test.

The following secondary endpoints had missing values: Inotrope Score post-bypass (Group-1 2, Group-2 2, Group-3 1), Inotrope Score 24 hours post-operatively (Group-1 3, Group-2 2, Group-3 1), Inotrope Score 48 hours post-operatively (Group-1 3, Group-2 2, Group-3 2), Inotrope Score 72 hours post-operatively (Group-1 3, Group-2 2, Group-3 2), Inotrope Score Total (Group-1 3 , Group-2 2, Group-3 2), Length of ICU stay (Group-2 1), Length of Hospital stay (Group-2 1), Death (Group-1 7, Group-2 3, Group-3 2), Myocardial Infarction (Group-1 7, Group-2 3, Group-3 2), Stroke (Group-1 7, Group-2 3, Group-3 2), Revascularization (Group-1 7, Group-2 3, Group-3 2).

## Discussion

Myocardial preservation during cardiac surgery is certainly one of the most debated topics in this field. One method of achieving myocardial protection is by using a cardioplegic solution administered into the heart to achieve a temporary arrest of the myocardium whilst the surgeon performs the operation in a bloodless field.

In the UK a variety of myocardial protection strategies are used, including cold blood antegrade cardioplegia with topical cooling, warm blood antegrade cardioplegia, warm blood antegrade and retrograde cardioplegia, cold blood antegrade and retrograde cardioplegia and cross clamp fibrillation.

In current practice the route of delivery is at the surgeon’s discretion and as such there is no consensus on using a specific route to supply the cardioplegia into the myocardium.

The most common technique used by the majority of cardiac surgeons is the antegrade route, in which cardioplegia is delivered into the aortic root and spreads via the coronary arteries supplying the myocardium. Although significant clinical evidence favours the safety of this method, severe stenoses of coronary arteries in patients undergoing CABG may prevent the uniform distribution of cardioplegic solution through the myocardium [23] and, importantly, sub-optimal or inadequate distribution to parts of the myocardium increases the risk for PMI.

A proposed solution to overcome this limitation is the retrograde route of delivery, in which cardioplegia is administered through the coronary sinus and through the extensive venous network of the myocardium. Following the pioneering idea of Pratts [24] who suggested that an ischemic myocardium could be revived back into its healthy form by supplying oxygenated blood through the veins, retrograde cardioplegia was applied for the first time by Blanco et al. [25] in 1956 and further developed in a significant number of centres: one of the seminal works on retrograde cardioplegia was conducted by Menasche et al. [26] who demonstrated that, during aortic valve replacement (AVR) surgery, post-operative haemodynamic stability, cardiac outputs and right and left ventricular stroke indices were similar in patients receiving retrograde cardioplegia compared to those receiving antegrade cardioplegia. The same authors also conducted a retrospective observational study on a relatively large group of patients undergoing isolated AVR or CABG surgery with or without concomitant valve surgery using retrograde cardioplegia alone [27] and, although there was no antegrade cardioplegia group for direct comparison, they reported that the overall trend in mortality rates was either similar or less than what other studies had shown.

With the knowledge that the anterior cardiac veins supplying the RV are not directly connected to the coronary sinus and thus may lead to a suboptimal distribution of the cardioplegic solution to the RV [12],[13] Kaukoranta et al. [28] conducted a small study on patients undergoing CABG surgery and receiving either antegrade or retrograde cardioplegia and reported that, despite more significant ischaemic changes within the RV in the retrograde cardioplegia group, no post-operative complication related to the retrograde route was observed.

It is therefore clear from the literature that multifactorial clinical endpoints have been used to determine a difference between myocardial protection strategies: moreover the population size in these studies was often too small to come to a meaningful conclusion on the benefit of a particular protection strategy. Importantly, many of the studies on retrograde cardioplegia used retrograde cardioplegia alone when compared to antegrade cardioplegia and only very few studies have compared the combination of antegrade and retrograde techniques against antegrade cardioplegia alone: this is one more reason to interpret current literature with caution.

In the only other study similar to our retrospective analysis, Radmehr and his colleagues [13] compared the combined antegrade and retrograde versus antegrade cardioplegia alone in patients undergoing CABG surgery and found that the use of combined antegrade and retrograde cardioplegia was associated with a 16.5% statistically significant decrease in inotropic requirement.

Our retrospective study suggests that myocardial protection can be improved by combined antegrade and retrograde technique and, in contrast to literature already available, our patient cohort is divided into three groups (the combined technique of antegrade and retrograde cardioplegia, antegrade cardioplegia alone and cross-clamp fibrillation). We only included patients undergoing CABG surgery and excluded subjects undergoing either valve surgery alone or CABG combined with valve surgery. This enabled us to assess the drawbacks of the antegrade technique via blocked coronaries and therefore the potential benefit of retrograde cardioplegia.

In addition to this, with an increase in the cross-clamp time the general understanding is that the myocardium is more prone to becoming ischaemic and damaged: our study suggests that despite longer cross-clamping times in the combined group, the total PMI was consistently lower in these patients when compared to the other two groups. This correlates with a retrospective study conducted by Bar-El et al. [29] on patients undergoing CABG surgery with or without valve repair or replacement and receiving antegrade followed by retrograde cardioplegia, demonstrating that the expected mortality was lower in patients with longer aortic cross-clamping times compared to those with shorter aortic cross-clamping times, and therefore indicating that retrograde cardioplegia can enhance myocardial protection despite the longer cross-clamp times. We therefore suggest that the absolute value of the cross-clamp time may potentially be less important than the type of myocardial protection used: this could be crucial in complex patients with poor LV function or anticipated long cross-clamp times, for whom the best myocardial protection available would be warranted.

Another important difference between groups 1 and 3 is the temperature employed during the cardioplegic techniques utilizing cold blood and warm blood cardioplegia respectively: the optimal temperature of cardioplegia during cardiac surgery is another crucial aspect of myocardial protection besides the actual technique used and it could be argued that the lower troponin rise in group 3 may be partially explained by the temperature difference. Cold cardioplegia is able to attenuate myocardial oxygen demand and the risk of ischaemic damage but conversely may lead to the inhibition of myocardial enzymes leading to a stunning of the metabolic and functional recovery following surgery. However warm blood cardioplegia is thought to counteract this potential deleterious effect. In a meta-analysis [30] involving 8814 patients randomised to either warm cardioplegia or cold cardioplegia predominantly in the setting of CABG surgery, there was no significant difference in all-cause mortality or incidence of myocardial infarction, intra-aortic balloon pump usage, stroke, low output syndromes and post-operative AF between the two patients groups and postoperative cardiac index was significantly improved in the warm blood cardioplegia group. Similarly, Tan et al. [31] compared cold to tepid cardioplegia and found no difference in mortality, peri-operative myocardial infarction, stroke or inotrope requirement.

Our retrospective study has several limitations. The cohort population was small and in particular the group consisting of the combined use of retrograde cardioplegia and antegrade cardioplegia contained only 10 patients, who were operated on by one consultant, which may result in some potential bias. Typically in a study of this type strong prognostic and confounding variables would be adjusted for in the analysis. In this case the small sample size precluded detailed adjustment and we acknowledge some residual confounding bias may remain. Finally, we have not adjusted for multiplicity in our analysis and there is a possibility that the results may have arisen by chance, and therefore the clinical outcome data will need to be confirmed in future studies.

In conclusion our retrospective clinical analysis suggests that the combined use of retrograde cardioplegia and antegrade cardioplegia during first time CABG surgery can be beneficial in reducing PMI. Also, importantly, to our knowledge there is no study which combines the following four factors together into one analysis on PMI: aortic cross-clamping times, combined antegrade retrograde versus antegrade alone versus no cardioplegia, hsTnT levels at 6 different time-points with AUC-hsTnT, and exclusively CABG patients. We do not suggest surgeons to change their practice for routine CABG surgery, as we have not yet demonstrated that the change in measured troponin levels may have a significant impact on clinical outcomes. However, we feel that this evidence should be available, so surgeons can choose to add retrograde cardioplegia for more complex cases in an evidence based fashion, knowing that the addition of retrograde cardioplegia may have the potential to enhance myocardial protection. Our retrospective study also suggests that larger studies are required in order to further evaluate our findings and to investigate whether the reduction of PMI in patients undergoing CABG would result in improvement of clinical outcomes.

## Conclusions

Our study suggests that, compared to traditional methods of myocardial preservation, the combined use of retrograde and antegrade cardioplegia may have the potential to reduce PMI in patients undergoing first-time CABG surgery. Whether this can lead to an improvement of clinical outcomes is yet unknown and therefore larger studies are required in order to further evaluate this potential impact.

## Authors’ original submitted files for images

Below are the links to the authors’ original submitted files for images. Authors’ original file for figure 1 Authors’ original file for figure 2

## Abbreviations

CABG: Coronary artery bypass graft
PMI: Peri-operative myocardial injury
AUC: Area under the curve
hsTnT: High-sensitivity Troponin-T
AKI: Acute kidney injury
ICU: Intensive care unit
AF: Atrial fibrillation
SD: Standard deviation
CK: Creatine kinase
CAD: Coronary artery disease
RV: Right ventricle
RIPC: Remote ischaemic preconditioning
PAD: Peripheral arterial disease
CPB: Cardiopulmonary bypass
