# Supplementary figures and images for: A retrospective analysis of myocardial preservation techniques during coronary artery bypass graft surgery: are we protecting the heart?
Source: J Cardiothorac Surg. 2014 Dec 31;9:184. doi: 10.1186/s13019-014-0184-7 (PMC4301898; doi:10.1186/s13019-014-0184-7)

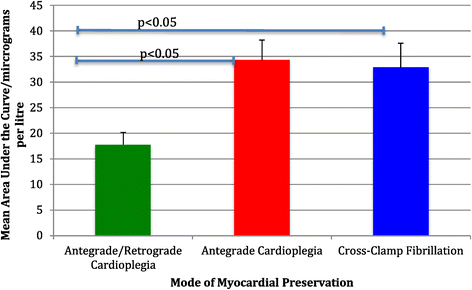

Supplement: Supplementary file 1 — Authors’ original file for figure 1 [file 13019_2014_184_MOESM1_ESM.gif]

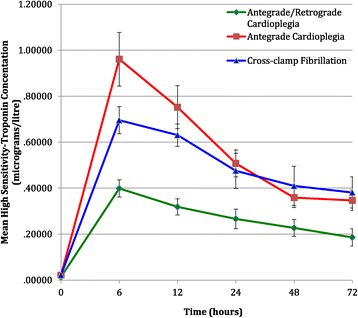

Supplement: Supplementary file 2 — Authors’ original file for figure 2 [file 13019_2014_184_MOESM2_ESM.gif]
